# Annual Address before the Medical Society of North Carolina

**Published:** 1872-02

**Authors:** Wm. A. B. Norcom

**Affiliations:** Edenton, N. C.


THE GEORGIA

MEDICAL COMPANION:

A MONTHLY ADVISER.

VOL. 2. ATLANTA, GA., FEBRUARY, 1872. NO. 2

### PART I.

### Original Communications and Special Selections.

### MODERN TREATMENT OF ACUTE INTERNAL INFLAMMATIONS.

BY WM. A. B. NORCOM, M. D., OF EDENTON, N. C.

*The Annual Address delivered before the Medical Society of the State of North Carolina, at its Fifteenth Annual Meeting, held at Warrenton, May 20th,* 1868.*

MR. PRESIDENT AND GENTLEMEN OF THE SOCIETY:

I must first thank you for the honor you have conferred upon me; and when I see before me so many, some whose minds are strong with the rich experience of years, so much more able to fill the position I occupy, my thanks are doubly due. I am glad to see that our Society, for several years unavoidably prostrated, is so gloriously reviving, and the large attendance at this meeting attests the interest felt by its members in its aims and objects.

Within a comparatively brief period of time great progress and real improvement have taken place in our profession, and many erroneous views and opinions, formerly held, have yielded to the

* Published by request of the Society.

advance of science and the development of truth. Nowhere is this more apparent than in the treatment of Acute Internal Inflamma-tions, and as they form no inconsiderable portion of the diseases we are called upon to treat, and some of them of so grave and serious nature, I have thought it not inappropriate on this occasion to inquire into what modern medicin has done for their treatment.

Foremost in importance stand the enforcement of the study of their natural history, and revival of the Hippocratian doctrine of a greater reliance upon nature rather than of a perturbatory and lowering treatment, and the substitution of a proper and sufficient alimentation for a low diet—yes, I may say, starvation.

Upon these points I shall chiefly speak, as I believe a proper appreciation of their importance, and their observance, indispensable to successful practice. Otherwise medicinal agents would be of little or no value. I shall then speak of a practice, which, unfortunately, is pursued by many even in this enlightened day.

Nothing so thoroughly arms a physician for his contest with disease as a knowledge of its natural history, that he may be prepared to imitate and assist the curative changes nature so constantly strives to effect. Our remedies can only avail in so far as they aid natural operation.

In a lecture delivered by Sir Wm. Ferguson,* at the Royal College of Surgeons, of England, in June, 1865, I find the following:

"The loss of confidence in much-vaunted remedies seems, in some respects, like a loss or diminution in our appliances—an abstraction from our powers, as it were. But in my opinion the correct view to take here is, that we are acquiring a knowledge of our own ignorance—that we are beginning to see that we have placed our faith erroneously. In short, we have been taking honor to ourselves for that which has been justly due to nature. We begin to see the difference between blind empiricism and natural processes."

Says Anstie,† on this point: "Without an observation of natural processes no medical man ever did great things for mankind, or for the advance of his art." * * * * "It was but yesterday that disease was universally regarded as something entirely foreign to the vital organism, which came to it from without, resided in it for a time, and then departed, exorcised by the physician's art. To-day we are inclined to take a less exalted view of our functions, to confess ourselves to be but the humble assistants in those curative processes which nature herself initiates, and very often carries through without our help, or even in spite of

---

* Richmond Med. Jour., vol. 1. p. 37. † Stimulants and Narcotics.—*Anstie.*

our ignorant interference. Together with such changed ideas there must come a revolution in our modes of therapeutical inquiry; and notably, a disposition to compare those instances of the beneficial action of drugs which are well authenticated, with similar effects *produced by the unaided operation of natural causes.* And it is surely lawful to hope that even partial success in this direction may prove more advantageous to the progress of our art, than the most brilliant reasoning which should presuppose the physician's power to effect radical alterations in the working of the vital agencies, whose operations we are only just beginning, dimly and partially, to understand."

And says Prof. J. Hughes Bennett: * "If every young practitioner would dedicate his life to the careful elucidation of the natural progress of only one disease, he would do more for medical practice than has been accomplished by centuries of empirical trials of remedies."

And Dr. Todd, † one of the brightest medical lights England ever produced, remarks thus: "Internal Inflammations are cured, not by the ingesta administered, nor by the egesta promoted by the drugs of the physician, but by a natural progress as distinct and definite as that process itself of abnormal nutrition to which we give the name of inflammation. Our interference either may aid, promote, and even accelerate this natural tendency to get well; or it may very seriously impair and retard, and even altogether stop, that salutary process."

Prof. T. Gaillard Thomas, ‡ in a lecture to his class, in speaking of the natural history of disease, says: "Remain ignorant of it, and you shut the gates of the avenue which leads to progress in medicine; master it, and your therapeutic knowledge will become certain, and its application a science." He further says, in the same lecture, as the result of experience: "If fifty cases of pleurisy (the disease for which Sydenham prescribed so vigorously,) be placed in bed, carefully nursed, dieted, guarded from deleterious influences, and receive not a particle of medicine of any kind, the probabilities are that not one case would end fatally; all would likely recover, unless some peculiarity of constitution, the unfavorable age of the person, or accidental application should alter the result."

How very liable we are to be deceived in regard to the power and efficacy of drugs! Suppose in these fifty cases of pleurisy some harmless medicine had been given! It might have been pro-

* Bennett's Practice. † Clinical Lectures, by Beale.

‡ Richmond Medical Journal, vol. 1.

claimed as a specific. Or, suppose mercury, so freqnently given by many physicians in this disease, had been administered to these patients, they may all still have recovered, but certainly not so quickly, nor so well as under the conditions mentioned by Dr. Thomas.

And says the popular and accomplished Professor of Physiology, Hygiene and General Pathology, in the University of Maryland:* "Now we substitute for the old compound prescriptions, simple remedies, given with a definite object, always recognizing the true position of nature as the curer, and medicine as her handmaid and assistant—laying great stress upon the observance of hygienic and sanitary laws."

Dr. Garrod† tells us that he has seen many cases of severe rheumatic fever get rapidly well without the use of drugs, and that on simply colored or camphor water the improvement is often very quick and decided. In the Guy's Hospital Reports, for 1865,‡ are forty-one cases of rheumatic fever, thirty-seven treated by Dr. Gull, and four by Dr. G. O. Rees, "scarcely any medicine except mint-water being given." Twenty-two were males, nineteen females; two only above the age of forty, the rest under thirty-five. The heart is mentioned as implicated in a large number of them. The average number of days from admission into hospital to complete convalescence was, for the males, sixteen, females, twenty-one. The average duration of the acute symptoms in seven cases, in which there was no evidence of the heart being involved, was eight days; in six cases, in which the heart was decidedly affected, twenty-three days. From these cases what other inference can be drawn except that mint-winter is a wonderful remedey for rheumatism, or that nature frequently triumphs over the disease? As mint-water is known to be inert, we must accept the latter. Such facts as these should teach us a wholesome lesson. Suppose these cases had been salivated! Modern authors tell us that salivation neither shortens the duration of the disease, nor prevents cardiac complications. Then why practice it when, to say the least, the convalescence would necessarily be prolonged by the patient having to get well of the treatment as well as the disease? I mention this treatment particularly because I know that by many mercury is considered the "heroic" remedy in this as well as all other acute diseases.

By the foregoing remarks, I would not be understood to advise doing nothing for rheumatism; but we might learn the lesson to be careful in the use of powerful drugs, when the unaided powers

---

* Valedictory Lecture, delivered March 9th, 1857, by Prof. Donaldson.
† Reynold's System of Medicine, vol. 1. ‡ Op. Cit.

of nature frequently effect a cure. In this disease the alkiline treatment has proved highly successful.

A short time since a lady, of great intelligence, told me that a few years ago she was treated for a pneumonia, the basis of which was *verat viride.* and low diet. Her medical attendant, a highly respectable physician, told her his object was to nauseate her to reduce her fever, which he very effectually did. She became cold, clammy and almost pulseless. Active stimulation had to be resorted to, to save her from immediate death. She, however, finally recovered after a very tedious convalescence of six or eight weeks.

From an authentic source I heard of a case that occurred within a few years past of bilious fever, which was terribly salivated. So offensive was the odor caused by the mortification consequent upon such ignorance and brutality, that one could not remain long in the room without sitting by a raised window. Yet this patient recovered, also, and was assured by the doctor that he would not have produced such a state of things had it not been "necessary to save life."

Who can say that these remedies aided those patients to get well? A faithful report of the *convalescence* of such cases would be most interesting and instructive. Do not such recoveries, gentlemen, furnish a crowning demonstration of the great fact that nature often triumphs over the doctor and his treatment too? What wonderful wisdom and goodness is displayed by the Almighty in permitting this to be so! If those only recovered who were properly treated, the inhabitants of this earth would grow less almost as rapidly as by a fiercely waged universal war. Call it what you may, the "*vis medicatrix naturæ*," or, as Dr. Dickson says, life itself, there is a resistive force—an inherent curative power—that frequently thwarts, in the language of Dr. Thomas, "the machinations of misguided men."

Dr. Forget* says, that "Nature is stronger than physic and physicians, for if she were the slaves of systems, the world would soon be a desert."

It is only by a careful and faithful study of her laws that we can hope to render to our patients that rational and effective aid, which it should always be the aim of the honest and conscientious votaries of our heaven-born art to give!

But let us pass on to Alimentation. It has always seemed strange to me that nutritious food, so essential to maintain the organism in its integrity, should ever have been withheld in disease, at which time it is now proved to be so indispensable. When

---

* North Carolina Medical Journal, October 1860.

nature is struggling to effect certain objects, which cannot be effected by art alone, and without which recovery could not occur, how very reasonable that we should give her the aid afforded by this agent! The system, worn and wearied by disease, and the blood impoverished, needs support and repair; and food, suited to the powers of digestion and wants of the system is, above all others, the remedial means suggested alike by science and common sense. If the position sought to be established by Dr. Chambers be correct, there can be no question of the propriety of food at the very inception of disease. It is this:* "That disease is, in all cases, not a *positive existence*, but a *negation;* not *a new access of action*, but a deficiency; not a *manifestation of life*, but partial death; and therefore that the business of the physician is, directly or indirectly, not to *take away* material, but to add; not to *diminish function*, but to give it play; not to *weaken* life, but to renew life."

I beg leave to quote a few extracts from a paper on "Alimentation in Disease," by Prof. Austin Flint, Sr., † read before the "Medical Society of the county of New York," Jan. 6, 1868.

Says he, "Certain it is that diseases which do not compromise directly the function of either the heart or lungs, cannot kill so long as the nutrition of the body is maintained at a point compatible with life. Starvation, associated with disease, may be inevitable; that is, the disease may involve an insuperable obstacle to either the ingestion of aliment, or its assimilation. Then it is that, in the language of Chossat, inanition may reach its termination sooner than the disease. On the other hand—and here is a fact full of practical import—starvation may not be a necessary effect of the existing disease, but may be due to insufficient alimentation. In such cases, inanition may prove a cause of death when the disease need not have destroyed life; the patient, indeed, may die of starvation notwithstanding the progress of the disease *per se* be favorble. Then, in the language of Chossat, inanition 'reaches its natural termination later than the disease which it covertly accompanies, and it may supercede the disease of which, at first, it was merelv an incidental element.'" * * * * "In acute diseases the failure of the vital powers is forestalled in proportion as nutritive supplies are assimilated. This is simply saying that the assimilation of nourishment is indispensable for the preservation of the powers of life. And then, in the progress of an acute disease, more or less failure of the vital powers ensues, the more nutrition can be maintained the more efficient the support." He further

---

* New York Medical Journal, Feb. 1868. † Renewal of Life.

‡ New York Medical Gazette, January 11, 1868.

says, that "Convalescence is protracted by the continuance of a liquid diet, and by an insufficient alimentation." Professors Barker, Jacobi and Noyes,‡ stated that their experience corroborated Dr. Flint's views, and Dr. Jacobi said, that "In children starvation is a very common cause of death, rather than the disease from which they are suffering."

In the "American Journal of Medcial Sciences," for January of this year, in an article written by him on "Inflammation, its Nature and Purposes," Dr. Jackson, in speaking of pneumonia, says: "Where the constitution of the patient is good, little more is required than to watch the course of the disease; the inflammation will take care of itself. It is the patient himself who is to be carefully looked to; his forces, which are to carry him through the conflict, are to be judiciously sustained, and all disturbing causes, moral and physical, guarded against. In cases of pneumonia, and where the antiphlogistic treatment had been fully carried out, convalescence is difficult and protracted. I have known two deaths to occur evidently from exhaustion. A limited portion of a lung had been the seat of disease, and was nearly restored to its natural state, and yet death took place with the disease extinct. Prof. G. B. Wood, says there is reason to believe that, in pneumonia, patients have been starved."

At the opening of the medical session at University College, London, Oct. 1, 1867, Prof. Graily Hewitt delivered the introductory address on "Nutrition the basis of the treatment of Disease." I will make from it a few quotations bearing directly on this subject. "But do we adequately recognize it as a fundamental principle in the treatment of disease that food is the most powerful of remedies? * * * * * The prescription may be otherwise faultless, its different ingredients balanced to a nicety, but the life itself must be supported and sustained, and this cannot be done without food.

"* * * * With some few exceptions, death is always preceded by exhaustion. The natural forces of the body become weakened in some way or other; another step downward, and the body ceases to live. Its mechanism is sometimes so disturbed or disarranged that resuscitation is in no way possible; but the mechanism being intact, the restorative power of food is great to an almost incredible extent. Nature herself frequently suggests the remedy, calls loudly for food, and will not be denied. The indication is then plain enough. But where exhaustion is great, appetite gone, consciousness itself, perhaps, well nigh extinct; it is under these circumstances that a knowledge of the extraordinary remedial action of nourishment is of vital importance. To place within the alimentary tube something which it may easily take up, and which the body may, with what little power is still left to it, convert into new force—to do this at the right moment, and in the right way is often an exercise of consummate skill and ability. The body is enabled thus to retain its hold on life. The deadly coldness gives place to genial warmth, the flickering pulse becomes steady, the light anew sparkles in the eye; for a time, at all events, the bitterness of death has passed."

Dr. Jackson once told me he thought there ought to be a Professorship of the culinary art in every Medical College, and that if physicians studied less about drugs, and more about alimentation, and the proper preparation of food for the sick, the result would tell decidedly for good upon their patients. Gentlemen, there is truth in this! How often are patients terribly drugged for diseases which could have been speedily cured simply by the observance of hygienic or dietetic rules!

The late lamented Velpeau frequently remarked in his lectures that more than half the people who got sick thought they must have medicine to get well; and what is worse, as great a proportion of doctors thinks so too. I think it was Schonlein, who said: "Good physicians often see no indication for treatment, bad ones never."

I conceive it to be our duty never to give medicine when it can possibly be dispensed with; and when needed, we should not give such as by lowering vital power, will materially interfere with nature's curative process. I would not have you think me a disbeliever in the propriety and efficacy of medicines, timely and properly administered, but secondary in importance are they to food and hygienic laws.

Dr. John Clark, in a single hospital, saved more than sixteen thousand children's lives by ventilation. What drugs could have done this? Just think, gentlemen, of the remedies that, from time to time, have been prescribed in typhus and typhoid fever!

In the lecture alreadly referred to, Dr. Thomas tells us that a few years ago the Commissioners of Public Charities in New York, assured by the physicians of Bellevue Hospital, that pure air and nourishment were the proper remedies for these diseases, entrusted their management to Dr. A. L. Loomis, of that city, (the patients being placed in tents on an island in East river,) he thus writes to Dr. Thomas concerning them: "I have had charge of the typhus fever cases for five months; during this time, not a particle of medicine and no stimulants have been employed, and the results have been one death in every sixteen and two-third cases; while, as you are aware, the per centage under the old plan was one in six. Dr.

Murchison, a late English writer, states them, in England, as one in five." Medicine has never been able to do this. I mention these facts, not to show the inutility of drugs, but the far greater importance of hygienc laws.

While the importance of ventilation is admitted by all, it is seldom properly practiced, and not unfrequently altogether neglected.

In regard to alcoholic stimulants, they are always indicated when there is failure of the powers of life, and may sometimes be given very largely to great advantage. Numerous authors relate cases that show their great value, and some in which very large quantities were given with decided benefit to the patient. I once gave an adult patient with a severe and extensive double pleuro-pneumonia, very nearly a quart of whisky every twenty-four hours, for two weeks. During the whole of this time very little food was given, owing to the inability of the patient to retain it. Perfect recovery occurred, but in consequence of the previous bad health of the patient, and the extent of the disease, it was slow and protracted.

But, gentlemen, (lamentable is it to know !) there are those who, either ignorant of, or disregarding the golden truths and facts of modern medicine, cling to tradition and views long since utterly exploded, and vaunt the success of a practice opposed to physiology and pathology—yes, it seems to me, to common sense. I refer to the so-called anti-phlogistic treatment, which originated long " ere modern physiology rent the veil of therapeutical empiricism," and the fatality of which is leading daily to its abandonment. This practice has no basis but tradition and empiricism. Scientific practice must have physiology for its basis. In the language of George Harley: * " A knowledge of organization, important though it be, is yet less indispensable to the physician than a knowledge of healthy function, for it is the latter which elucidates the dark problems of life,—it is the latter which proves the golden key to the comprehension of disease." And says Chambers :† " Is it not then obvious that the only sure mode of arriving at a knowledge of the deficiencies of vital powers, or diseases, is by a knowledge of those powers of which they are deficiencies ? The physiologist is the only true pathologist." And in part 1, of Todd, Bowman and Beal's "Physiological Anatomy and Physiology of Man ;" the latter says : " Pathology is the physiology of disease ; and it is obvious that no pathological doctrines can command confidence, which are not founded upon accurate views of the *natural* functions. It is also certain that im-

* Harley on Jaundice. † Op. Cit.

provements in pathology must follow in the wake of an advancing physiology."

Just here I will quote a little passage from Habershon, on "the injurious effects of Mercury." Says he: "Any remedy that has been supposed to possess properties by which this so-called inflammation could be checked, has received the name of antiphlogistic, and mercury stands foremost amongst them; but water or brandy often fulfill a similar purpose, and many agents possess equal power in this respect. This phraseology is a vestige of days of ignorance, and has only hypothesis to rest upon. In medicine, however, we still retain the antiphlogistic remedy; and too often diseases are considered as conditions requiring to be smothered out, unfortunately also, by frequently extingushing the patient."

But let us examine more particulary into this subject, and see how irrational such practice is. At the present day, I am sure there could be found no bleeders like Clutterbuck, Rush or Dewees, yet there are those who believe in the *curative* agency of the abstraction of blood by venesection. Its mechanical effects for good no one will deny; yet, even here, Dr. Chambers tells us that the loss of this "liquid flesh" must be immediately compensated for by the administration of nutritive food; but it is indeed difficult to understand how venesection can exercise a *curative* effect upon the part inflamed. The attempt to cut short an inflammation by a large bleeding, early resorted to, is now so little practiced, that I will not speak of it.

The question naturally arises, can general blood-letting diminish the amount of blood in the part inflamed? Yet this is not always necessary, as in pneumonia, where the cure is effected by cell-growth, to accomplish which an increase amount of blood is necessary. In inflammation, in consequence of injury to the *vaso-motor* nerves of the part, the vessels lose their contractile power, and become distended with blood, and stasis, owing to adhesiveness of the corpuscles, occurs, followed by exudation. No one pretends to say that general blood-letting can directly diminish the amount of blood in the part inflammed in external inflammations, and why should it in internal? It can only be done by such a large loss as would materially and alarmingly weaken the force of the heart, and then not more would probably be taken from the inflamed part than would be by the application of a single leech to an external inflammation, and all this time the inflammatory process goes on unaffected by the loss of blood. In the language of Markham, "venesection is not a remedy for inflammations, but for the accidents of certain of them." In a word, it acts mechanically, and in this way may sometimes prove benficial.

In pneumonia, it is the custom with not a few to abstract blood locally by cups and leeches, when the anatomist shows that there is no direct anastomosis between the surface vessels and those of the inflamed part. Hence, it is plain that cupping or leeching the foot or back of the neck would do as well, so far as the loss of blood is concerned. Local depletion can only be beneficial where there is direct vascular connection between the surface from which blood is drawn and the part inflamed. When this does not exist, the good effected by such means is only through reflex action upon the vaso-motor nerves of the part.

Those who bleed on antiphlogistic principles do not do so to the extent practiced by their predecessors, and the majority of them are generally ready to assign as the reason for this, that these diseases have changed their type—that we are at present on an adymamic, asthenic, tide—and patients cannot bear the same losses of blood now as formerly. In this view some moderns agree with them. If this be so, it is passing strange that this change from strong to weak should have occurred nearly all over Europe and this country about the same time. The surgeon now-a-days does not say this about external inflammations; and if his patient should die from loss of blood, or a woman from excessive flooding after labor, he does not in the one case, not the accoucheur in the other, invoke the change of type theory as a cause. And when large bleedings are now practiced the same fatality occurs as formerly.

Mercury is not so lavishly given now in these diseases as in the past, yet by some it is not administered with a *very* sparing hand. Why is not the change of type theory invoked as a reason for this? But, gentlemen, it is not my purpose to discuss this subject; the real and true cause, however, of our change in pratice is a better knowledge of these diseases, resulting from advance in pathology and improved methods of diagnosis.

But within the past few years there seems to be an abatement of their sanguinary propensity in those even who bleed on purely antiphlogistic principles in acute inflammation, and they have betaken themselvers to other remedies scarcely less powerful for evil. I refer to Mercury and Tartar Emetic. Let us inquire into the action of these agents, particularly the former, and see if they produce a condition favorable to the object in view, and are sanctioned by the authority of those who have had the best opportunities of testing their merits.

"Mercury," says Headland,* (accepting the experiments of Wright,) "by some destructive agency, deprives the blood of one-third of its fibrin, one-seventh of its albumen, one-sixth or more of its globules, and at the same time loads it with a fetid matter, the product of decomposition. Such power is possessed by few other medicines, and certainly exerted by none in the same degree as Mercury. It is an agent of terrible activity, and we may well be cautious how we handle it. Mercury wastes the frame, causes the body to become thin and feeble, the face pallid, and diminishes the nervous energy," And Habershon says: † "After mercury has been taken for some time, the general nutrition of the body is impaired, the blood becomes darker, the coagulation of its fibrin less firm, and the proportionate quantity of serum increased, the red cupuscles are diminished, and the patient becomes thin and blanched. His tissues lose their proper tone, his muscles become flaccid, his energy diminished, and his nervous system enfeebled." And while admitting that serious, and abnomal deposits sometimes become absorbed under its influence, he further says "there is ample proof that the same can be effected by less injurious means, and that it sometimes happens that the diseased product becomes more abundant in quantity and less organized in character from the enfeebled nutritive action consequent on the mercury." He also says, in acute pleuritis, pericarditis or peritonitis, it is the ordinary practice to give calomel so as to affect the gums, but that the disease often subsides without any mercury, and very frequently the effusion steadily increases during salivation. He states that he has seen cardiac disease, consequent upon rheumatism, come on while the system is under the influence of mercury.

Tanner, in his work on the Practice of Medicine, says: "With regard to the use of mercury, there appears to be every reason to believe that its utility in controlling inflammation, or in promoting absorption of the effused products, has been very much overated; and indeed it seems highly probable that inflammatory diseases will progress more favorably without the use of this medicine than with it."

The cases of pericarditis published in the London Lancet about twenty years ago, treated by Dr. John Taylor, without mercury, show the undeserved reputation this medicine has had in this disease, and subsequent observations by others confirm his results.

Dr. Todd says: ‡ "No one would now venture to assert that mercurial influence, however quickly induced, ever checked pericarditis or pleurisy; nor would it be easy to adduce an instance in which, with any reasonable degree of certainty, it could be stated that mercury broke down adhesions, or prevented their occurrence."

---

* Action of Medicines.

† Injurious effects of Mercury.

‡ "Clinical Lectures" by Beale

Dr. Garrod, whose views are entitled to great respect, in treating of rheumatism, says: * "For many years I was in the constant habit of administering calomel in cases in which inflammation of the heart was present, but for the last eight or ten years I have not done so as frequently, and have seen no reason to regret the change of practice, the cardiac inflammation appears to have yielded quite as readily, and the patient, on the subsidence of the fever, has not had to suffer from ptyalism in addition to debility."

One single case of rheumatic fever † in which pericarditis came on while the patient was salivated, and proved fatal, seems to have caused Dr. Chambers to discontinue its use in this disease; and in pneumonia he says that antimony and mercury, are "pure destructives," "merely abet the worst effects of the disease."

Prof. Bennett and a great number of other modern scientific physicians, as strongly condemn this destructive agent as those from whom I have quoted. So small a proportion does the good bear to the ill effected by the administration of this drug in these diseases, that it would doubtless be better for the human race if its use in them could be entirely interdicted. Perhaps it may sometimes be used to advantage, but the deliterious results which follow its misuse, to be so frequently seen too, are enough to make the conscientious physician look, with a scrutinizing eye, for its real virtues. No remedy is more generally abused. In the malarial sections of this State few persons can be found who, for a slight attack even of fever, do not think a dose of calomel or blue mass indispensible to "set the liver right," as they say, when quinine alone, or sometimes, perhaps, aided by some mild and gentle means, would be amply sufficient to effect the cure.. The prostrating effects of such a course, aided by low diet, renders them prone to renewed attacks, which generally follow, and the autum finds them weak, feeble and anæmic, and their blood loaded with black pigment. Should their vocation cause them to be much exposed in inclement weather, acute disease, probably pneumonia, attacks them, and the grim messenger, frequently sent to end their existence, doubtless thanks mercury for its timely and efficient aid in his work of destruction.

But I am digressing. I must pass on to my subject, and shall say a few words only in regard to tartar emetic.

As a depressant, to lower the force of the heart, in the early stage of acute inflammation, this drug, though much less than formerly, is still prescribed after the plan of Rassori and Læennec, though in not so large doses, not only by the adherents of anti-

* Reynold's System of Medicine, vol. 1. † Op. Cit.

phlogistic principles, but by some modern practitioners. The b st success ever gained in the treatment of these affections has been by well-directed and persistent efforts to *sustain* rather than depress the heart's action, and the total avoidance of depressing agents. Why depress the heart's action when it has been already done by the disease? And besides, the nausea occasioned by this remedy prevents the administration of food. Dr. Flint, in the paper from which I have already quoted, says: "Medicines not unfrequently impair the appetite and interfere with digestion. If not required for a special curative effect, they are then likely to do harm by compromising, more or less, alimentation and nutrition." When in the early stage of these affections pain and dyspnœa imperatively demand relief, and the functions of the heart and lungs seriously impeded, small bleedings from the arm may be practiced on *mechanical* principles, and when these are not admissible from fear of ulterior ill effects, antimony would not be a proper remedy. It is one of those destructive agents which Chambers calls, with Mercury, in pneumonia, a poison, and we cannot be too careful in its administration. Even Headland, who seems very partial to both antimony and mercury, says: * "Antimony deteriorates and impoverishes the blood in very much the same way as mercury."

Veratrum viride, a powerful cardiac depressant, is used by many for the same object as antimony; yet I am disposed to think that the effect produced by such agents is antagonistic to the principles of treatment pointed out by a correct pathology. If Veratrum Viride, etc., diminish the *frequency* of the pulse and increase its *power*, as some assert, then they must be, like digitalis, cardiac *tonics*. *If so*, they are proper remedies.

A few months ago I treated a child eight years old with severe pneumonia—saw it twenty hours after the inception of the disease. At my first visit the pulse was 140 and respirations 70 to the minute. The treatment consisted in local warmth, an average of three pints of milk, one and a-half pints of rich soup with little alcoholic stimulus every 24 hours. No medicine was given except anodynes and diuretics. On the sixth day the child sat up by the fire, and on the tenth was dressed and walking about the house. I am sure this result would not have been accomplished by an antiphlogistic treatment—by depressing the little patient still more than had been done by the disease.

Let us bear in mind that there are no foreign forces to be attacked, nor is there an excess of vitality, but a deficiency of the

---

Action of Medicines.

powers which naturally reside in organism. Indeed it may be that the cause of the attack which demands our aid is an already deficient vitality. I am every day more and more convinced that a recognition and observance of these important facts must form the basis of successful practice. Rather than being too intent upon driving out the enemy, let us busy ourselves, as Dr. Bennett says, to secure the safety of the fortress—let us try to bring the individual *up* to his physiological status. In a word, let us help him to restore his *natural* powers. This support can only be given by food. As Dr. Hewitt says: "Nutrition is the basis of the treatment of disease, and no other is possible for a rational system of medicine."

In the preface to his admirable little brochure on Hysteria, Mr. Skey says: "A week condition of the animal body is intelligible enough, but an abnormal condition warranting a reduction of vital power by artificial agency, I cannot understand." Let us construct and support, not destroy and weaken.

The experiments of Hering and others show that in pathological increase of the heart's action the rapidity of the general circulation is generally diminished. And M. M. Estor and St. Pierre, have shown that the venous blood returning from an inflamed part is of a brighter color than ordinary venous blood, showing *suboxidation*. These facts certainly do not call for depressing agents in the treatment of inflammation. On the contrary they show diminished life. And besides, the general condition of the patient strongly indicates a lowered vitality. The least exertion frequently cannot be borne even at the very inception of disease, and that which would be slightly prejudicial to the normal life would very seriously affect the pathological state.

How different the practice we condemn from the one we adopt —the Restorative and Eliminative. Modern medicine teaches us that these affections cannot be cut short, and that while we aid nature by the most nutritive food, and alcoholic stimulants when necessary, to bring about most important changes, we, at the same time, give such remedies as will assist in the removal of effete products by the emunctories. I refer, of course, to diuretics and diaphoretics. Rest in bed and support are necessary from the first; local warmth, local depletion, and blisters sometimes, are most important remedies. Expectorants, so frequently given in pneumonia, are not generally called for, as the exudation matter is in a very great part removed in other ways; and, too, they frequently cause nausea and thus offer an obstacle to alimentation. Cathartics, of course, are sometimes needed.

This practice is sneeringly denounced by some as "expectant."

In reply to this I will quote the closing paragraph of Dr. Chambers' article on pneumonia in his "Renewal of Life." "Doing nothing or leaving the patient to himself, would indeed be dishonest; but do we do so? Is it doing nothing to keep up constant relays of poultices night and day for a week or ten days? Is the enforcement of continuous nutrition no labor? Is there no anxiety and thought spent in hourly watching the need of variation in our doses of opium and wine for serious cases? Is the moistening and warming the air to an even temperature not enough to occupy our time? Is it so much easier to support the waning life than to weaken it, that the former should be condemned as idleness, the latter praised as activity? If the pneumonic patient were left to himself would he—could he—adopt any of the means suitable for his recovery? Would he not very likely be taking colocynth, senna, calomel, antimony, ipecacuanha, salines, senega, squill, hydrocyanic acid, colchium, be rubbing in mercury, applying mustard poultices, and blisters, be bled "*coup sur coup*," or have brandy every half hour? Is it nothing to stand sentry against the fatal seductions of polypharmacy?"

This treatment, gentlemen, simple as it may seem, and indeed really is, is practiced by almost all modern scientific physicians, and they tell us its success far transcends every other. In addition to the actual saving of life, convalescence is very rapid after the disease subsides.

It is our duty to shake off the shackles of tradition, if they fetter us, and walk in the light of *to-day.* It is no easy task to get men to confess that they have been practicing error, and to adopt a treatment contrary to the teachings of their years; yet, in an incomplete and advancing science like ours, the physiologist, histologist and pathologist are constantly furnishing us new facts upon which to build a more successful practice.

Just as Jenner startled the world with his great discovery,* Dr. Wm. Aspinwall had erected in his native town, in Massachusetts, small-pox hospitals, at great expense, for the accommodation of persons to be inocculated, from which he would doubtless have reaped a large fortune. He immediately acknowledged the great superiority of Jenner's discovery, though at a great pecuniary loss to himself. He remarked to Dr. Waterhouse: "As a man of humanity I rejoice in it, although it will take from me a handsome annual income." In this spirit let us study our profession, never forgetting Art. 2d of the Constitution of our Society.

---

*Introductory, by Prof. H. H. Smith, to the Class at University of Pennsylvania, Session '56—'57.

To Bernard, Robin, Beale, Bennett, Todd, Anstie, Chambers, and others, abroad and in our own country, we owe an impayable debt of gratitude, and errors are fast disappearing before the truths established by their patient and arduous labors. Let us not call them sceptics and innovators, but bold and fearless pioneers, worthy of imitation, in a noble work. The opposition with which they meet is like that which, throughout all time, has attended the introduction of anything new. One of the most learned and accomplished of American physicians has said: * "Every new fact and doctrine, adverse to those generally received, is repelled and rejected by those whose ideas and opinions have been firmly settled. There are too many interests involved—too much self-love wounded, and preconceived ideas to be destroyed, to admit of the prompt reception and adoption of new thoughts or facts, or ideas, either however true they may be." And Newton said: * "A man must resolve to put out nothing new, or to become a slave to defend it."

This is well expressed, also, by Prof. Draper, in an Introductory delivered by him in the "University of New York," in 1864: "It has been the experience of every age, that all attempts at reformation in pursuits from which extensive bodies of men draw their daily bread are destined to meet with a furious resistance. Try a reformation in any of these, and see what is the result. The living of the professional man is involved. You cannot expect that he will acknowledge that he has been practicing error all his days. These are no new principles—they were understood by one in old times as well as now. 'Put forth now thy hand and touch him in his substance, and he will curse thee to thy face.' To accomplish such changes it needs men of uncommon firmness and decision."

The old, especially, cannot easily free themselves from the baneful influence of routine—forsake views long cherished and accept modern truths. That great and good man, Prof. Jackson, of whom one of his eminent colleagues † once said, that he was "ever on the alert to take advantage of new lights which shed lustre on our profession," once beautifully expressed this in a letter to me. Said he: "Those advanced in years, whose opinions have been formed and acted on for years, almost invariably remain stationary, and repel all ideas, and even facts, that would disturb the peaceful stillness of their intellectual world." I am glad to say he is an exception to this.

Let us, free and untramelled, honestly strive after truth, not letting preconceived views operate as a barrier to its march, nor yet too prone to accept what is not sanctioned by clinical experi-

---

* Introductory by Prof. Sam'l Jackson, Sess. '55 and '56. † Prof. Leidy.

ence. In the language of Stokes: * "Above all things, follow truth; nature can never deceive—see that you be her faithful interpreter."

In some of the affections about which we have been speaking, a knowledge of Anscultation and Percussion is indispensable to a correct diagnosis, without which our treatment must of course, be worse than useless. This knowledge enables us to interpret the language of the heart and respiratory apparatus, and thereby, when possible, to relieve their wants. Without an acquaintance with Physical Diagnosis we can make no progress in this most important department of our profession. In speaking of the physical signs in health and disease, Da Costa says: † "Their importance for diagnosis it is difficult to over estimate. A knowledge of the physical signs is the solid foundation, without which any structure that may be raised will soon tumble to pieces." And yet how many physicians daily treat grave diseases of the heart and lungs, utterly ignorant of this most important aid to their diagnosis and successful treatment! I cannot think this would be so, did they properly appreciate the heavy responsibility resting upon them, and the sacred trust of which they are guardians. Justice to our patients demands from us a life of unremitting study and professional devotion. Ossified must be that physician's heart, and dead must he be to every sense of duty, who can see a wife or husband mourning over the loss of their best earthly friend, or the mother screaming for her babe, when he, entrusted with their lives, was ignorant of the means modern medicine had pointed out for their rescue.

In almost every department of medicine the same great progress has been made, to keep pace with which, though it requires incessant study, is yet our paramount duty.

And now my task is done, and poorly; yet, if I have awakened in any one of you an increased zeal in the study of our profession, or added to the deep responsibility which you all must feel, I shall have been amply rewarded for my humble effort.

Then, gentlemen, with hearts aglow with enthusiastic devotion to the noble cause in which we have enlisted, and minds deeply imbued with the love of truth, and uncontaminated and unimpaired by vice and idleness, let us renounce traditional teaching when it conflicts with the revelations of modern medicine, and let our labor be commensurate with the sacred responsibility and importance of our great mission, and we will become known and recognized, not only as medical practitioners, but scientific physicians—"the true interpreters of the laws and states of man's organism in health and disease, and the exponents of Positive Philosophy in Medicine."

---

* Practice of Medicine. † Medical Diagnosis.

